# Care of the Eyes

**Published:** 1883-12

**Authors:** 


### CARE OF THE EYES.

A GREAT deal of irreparable mischief is done by ignorantly meddling with the eye. The old rule "never to rub the eyes except with the elbows," is a little too radical to be cleanly, but it contains a useful hint as to caution. The eyes are delicate and sensitive organs, and so essential to our happiness and well-being that no one should neglect to guard them with the greatest care.

In serious troubles of the eyes a person should not neglect to seek promptly the advice of an experienced oculist. Great mischief has been done by quacks. We saw a case where a charlatan had applied a belladonna plaster to the eyelids of a lady whose eyes were only temporarily weak from a cold. She was directed to wear the plaster all night. In the morning she was blind, and never recovered her sight afterward.

Alum curds, poultices of all kinds, and even sponges or cloths, wet in warm water and applied during six or eight hours at a time, are extremely hazardous to the eyes. Even this apparently simple treatment may result in ulceration of the cornea and permanent loss of vision. The above facts are well known to competent physicians; but we venture to say that not one person in ten, not a physician, who reads this article ever knew before that a treatment so commonly resorted to in the family was fraught with such serious danger. It is well to keep the eyes cleansed with tepid water, and, in case of inflammation, a fine sponge wet with warm water may be applied half an hour or so at a time, but it would be better to consult a physician before doing anything. When the eyes are sore Nature provides a remedy in the warm tears that flow over them, keeping them warm and moist.

The cornea has no blood-vessels, but is nourished by imbibition; hence it has but little power to resist the action of warm fomentations, and the tendency of such applications is to soften and weaken it, so that if that treatment is carried too far the next stage is ulceration and destruction of the cornea and the sight.

We usually endeavor to give reasons for our statements, in order to impress important facts on the memories of our readers, particularly when treating of a subject of such general interest. More than half the cases of blindness have resulted from ignorance of the facts stated in this article; and still not one person in a hundred, who reads this number of the JOURNAL, and who is not already a subscriber, will invest one dollar in a subscription for the year 1884, though a single article might impart instruction, which if heeded would save the life of the one dearest to him.

